# Onset of Action of Azelastine 0.15% Versus Olopatadine/Mometasone for Allergen‐Induced Rhinitis: An EEU Crossover Study

**DOI:** 10.1002/clt2.70213

**Published:** 2026-11-08

**Authors:** Jean Bousquet, Ludger Klimek, Hans‐Christian Kuhl, Duc Tung Nguyen, Rajesh Kumar, William E. Berger

**Affiliations:** ^1^ Immunology and Allergology Fraunhofer Institute for Translational Medicine and Pharmacology ITMP Berlin Germany; ^2^ Institute of Allergology Charité—Universitatsmedizin Berlin Corporate Member of Freie Universitat Berlin and Humboldt‐Universität zu Berlin Berlin Germany; ^3^ Department of Otolaryngology, Head and Neck Surgery Universitätsmedizin Mainz Mainz Germany; ^4^ Center for Rhinology and Allergology Wiesbaden Germany; ^5^ Biometrics Meda Pharma GmbH & Co KG (A Viatris Company) Bad‐Homburg Germany; ^6^ Global Clinical Sciences MEDA Pharma GmbH & Co KG (A Viatris Company) Bad Homburg Germany; ^7^ Mylan Pharmaceuticals Private Limited (Now Viatris) Bengaluru India; ^8^ Allergy & Asthma Solutions Coto de Caza California USA

**Keywords:** allergic rhinitis, AZE 0.15%, Environmental Exposure Unit, nasal and ocular symptoms

## Abstract

**Background:**

The aim of this study was to compare the onset of action (primary), and overall efficacy (secondary) of azelastine nasal spray 0.15% (AZE 0.15%) with mometasone/olopatadine combination therapy (OloMom) in subjects with allergen‐induced rhinitis under controlled allergen exposure conditions in an Environmental Exposure Unit (EEU).

**Method:**

This was a randomized, double‐blind, placebo‐controlled, three‐period Williams cross‐over study. Participants were randomized to receive AZE 0.15%, OloMom, or placebo. Efficacy was assessed during two pollen challenge sections in an EEC, after a single dose and after 3.5 days of treatment. Following a minimum 14‐day washout period, subjects received alternate treatment and repeated study procedures until all three interventions were evaluated. The primary endpoint was time to onset of action based on change from baseline in Total Nasal Symptom Score (TNSS). Secondary assessments included total and individual nasal and ocular symptoms and overall efficacy after 3.5 days of treatment.

**Results:**

Eighty‐four subjects completed the study (mean age 41.5 years; balanced sex distribution), all with confirmed ragweed sensitivity. All treatments reduced TNSS post‐dose, with most effective and fastest relief from AZE 0.15%, onset at 15 minutes, sensitivity analyses supported onset at 10 minutes, and greater early nasal, ocular, and total symptom relief compared to OloMom and placebo.

**Conclusion:**

AZE 0.15% delivered rapid and clinically significant relief of AR, demonstrating greater early symptoms improvement compared with OloMom. Overall efficacy was comparable between treatments, supporting AZE 0.15% as an effective, steroid‐free alternative for rapid symptom control in patients with pollen allergies.

## 1 Introduction

Allergic Rhinitis (AR) is an IgE‐mediated inflammatory condition of the nasal mucosa triggered by allergen exposure [1]. The available therapeutic options for the treatment of AR include allergen avoidance, oral/intranasal antihistamines (INH), intranasal corticosteroids (INCS), leukotriene receptor antagonists, and allergen immunotherapy. According to the Allergic rhinitis and its impact on asthma (ARIA) Guidelines 2020 [2] and 2025 [3], intranasal antihistamines (INH) act rapidly, and combining INH with intranasal corticosteroids (INCS) is more effective than INCS alone. Antihistamines block H1 receptors, providing quick symptom relief [4]. Second‐generation antihistamines like azelastine (AZE) also exhibit anti‐inflammatory properties [5]. In contrast, corticosteroids reduce inflammation by inhibiting cytokine production but have a slower onset [6] although some fast non‐genomic effects may be present [7, 8].

Patients with AR prefer treatments with rapid onset and high efficacy to improve compliance [9]. Rapid relief from AR symptoms such as nasal congestion, sneezing, and itchy eyes is particularly desirable [10]. Azelastine hydrochloride is available in low‐dose (0.10%) and high‐dose (0.15%) formulations. The 0.15% version includes a taste‐masking agent, provided a stronger efficacy than the AZE 0.1% nasal spray [4], and offers flexible dosing. It is the only intranasal antihistamine shown to relieve both nasal and ocular symptoms for up to 24 hours [11]. A recent study showed that AZE 0.15% is safe with long‐term use over 12 months and improves RQLQ at 3, 6, 9, and 12 months similarly to intranasal mometasone [12, 13]. Controlled studies using Environmental Exposure Units (EEUs) showed that AZE 0.10% provided symptom relief within 15 minutes, outperforming oral antihistamines and INCS [13]. A newer combination spray, OloMom (olopatadine hydrochloride/mometasone furoate), demonstrated symptom improvement within 10 minutes [14]. Although AZE 0.15% has demonstrated rapid symptom improvement [12], comparative chamber data against active combination therapy remain limited.

The current study aimed to evaluate AZE 0.15%’s onset of action using an EEU model versus placebo and OloMom. Additionally, since mometasone's full effect takes up to 48 hours, a further aim of the study was to compare the overall efficacy of AZE 0.15% with OloMom after a management relevant period of 3‐day regular use, mimicking patients' treatment compliance behavior and when the maximum effect of mometasone containing nasal spray was expected.

## 2 Methods

### 2.1 Study Design

The study was a single‐center, randomized, placebo‐ and active‐controlled, double‐blind, three‐period crossover trial using William's design comparing azelastine 0.15%, placebo nasal spray (azelastine 0.15% vehicle) and fixed dose of mometasone furoate and olopatadine. It included a Screening Visit (Day −28 to −2) and a Priming Visit (Day −1), where nasal and ocular symptoms during a 2‐h ragweed exposure in an Environmental Exposure Unit (EEU) were recorded.

On Day 1 (Visit 3), eligible subjects entered the EEU and received a single dose (2 sprays per nostril) of AZE 0.15%, OloMom, or placebo 2.5 hours after exposure began. Symptoms were recorded over the next 4 hours using an electronic diary (ePDAT) and the assigned treatment was continued at home for 3 days (2 sprays per nostril twice daily). On Day 4 (Visit 4), the morning dose was administered at the site before another 4‐h EEU session with symptom tracking. After a washout period of at least 14 days, subjects returned for Periods 2 and 3, repeating the same procedures with alternate treatments. Minimum TNSS thresholds were not required for re‐entry, but nasal blockage was assessed before dosing. Any complete blockage was documented in the electronic case report form (eCRF). Throughout the study, investigators monitored and managed adverse events (AEs) appropriately.

### 2.2 Patients

Eligible subjects were males or females aged 18–55 with a history of seasonal allergic rhinitis (SAR) to ragweed for at least two seasons, a positive skin prick test, and willingness to complete all study visits. To be eligible for this study, asymptomatic or mildly symptomatic subjects (TNSS ≤ 3) at entry were required to have a TNSS ≥ 6 and runny nose score ≥ 2 at least twice during the 0–2‐h EEU period, with one occurrence in the last two time points.

Exclusion criteria included known allergies to azelastine, olopatadine, mometasone, or excipients; untreated nasal infections (e.g., herpes simplex); recent nasal surgery or trauma; history of anaphylaxis or significant systemic diseases; asthma diagnosis or FEV1 < 75% predicted; pregnancy, breastfeeding, or inadequate contraception; and use of prohibited or interfering medications. Subjects with complete nasal blockage prior to dosing were also excluded. All exclusions were assessed at the discretion of the Investigator to ensure subject safety and data integrity.

### 2.3 Sample Size Estimation

The sample size estimation was done for achieving an onset of 10 min. Previous studies showed that the effect of AZE can be expected in the range of 0.94 TNSS difference to Placebo at 10 min [10]. Assuming a treatment difference in change from baseline of 0.94 instantaneous TNSS points or higher and a standard deviation of intra‐individual treatment differences not higher than 2.5, 77 subjects were needed to achieve a power of 90% for the primary comparison of Azelastine 0.15% against Placebo at 10 minutes (Type I error of 5%, two‐sided). To achieve a balanced distribution to the 6 sequences of this 3‐fold cross‐over study and account for up to 8% dropouts not completing both periods for primary comparison (AZE and Placebo), further 7 subjects resulting in a total of 84 subjects were to be randomized in this study (14 subjects for each of the 6 sequences).

### 2.4 Study Duration

In this study the planned duration from screening (Visit 1) to end of the study was up to 83 days for every subject (duration may have been extended if re‐priming was necessary). Here, three treatment periods were included, each consisting of 3.5 days, with at least 14 days of the periods.

### 2.5 Ethics

The study was approved by Health Canada and conducted in accordance with the Declaration of Helsinki, ICH‐GCP (E6 R2), GDPR, and applicable regulations. Written informed consent was obtained from all participants, reviewed by an independent ethics committee (Advarra, approval notice no. MOD01880307), and documented in both the subject's and site's records.

### 2.6 Statistical Analysis

#### 2.6.1 Primary Analysis—Onset of Action

The primary analysis of this cross‐over study included data from all 3 treatments and from 3 periods (Visit 3, 6, and 9). Analysis of changes from baseline by each post dose assessment time point in 0–4 hours were performed using a mixed effect analysis of covariance model (MMRM ANCOVA) based on all subjects in ITT population. The model included treatment, period, and treatment sequence as fixed effects, and baseline as covariate and was run by time point. The covariance matrix over treatment periods was left unspecified. Degrees of freedom were determined by Satterthwaite approximation. Multiplicity of timepoints was handled by hierarchical testing starting from 4h post dose backwards. Tests were based on two‐sided type I error of 5%. In case of the active treatment, the onset of action was specified as the first time point after starting of treatment when the product demonstrates a greater change from baseline in the efficacy endpoint compared to the Placebo treatment that proved durable from that point until the end of the last assessed time point (4h; Visits 3, 6, and 9).

#### 2.6.2 Sensitivity Analysis

As sensitivity analysis, the PP set of subjects were a subset of the ITT population excluding the subjects with major protocol violations that may affect the results of the primary analysis. In addition, since the actual time points of symptom score assessments are critical for evaluation of onset of action, one sensitivity analysis was conducted to support the primary estimand in excluding all out‐of‐window (OOW) assessments. Multiple imputation method was used to handle missing data under the assumption of missing at random.

##### 2.6.2.1 Overall 4h Efficacy After First Dose

The overall change from baseline in TNSS, TOSS, T7SS, and individual nasal and ocular symptom scores for 0–4 hours after dosing (Visits 3, 6, and 9) were performed using all time points together in a ANCOVA model similar to analysis of primary endpoint additionally with time point as fixed effect (covariance matrix over periods and time points left unspecified).

##### 2.6.2.2 Overall 4h Efficacy After 3‐Day Treatment

The change from baseline in TNSS, TOSS, T7SS, and individual nasal and ocular symptom scores to 0–4 hours in the EEU session after 3‐day treatment (Visits 4, 7, and 10) were performed using same model as for overall 4h efficacy after dosing. The values of corresponding visit 3, 6, and 9 (i.e., before dosing) were used as baseline. All secondary endpoints were analyzed on ITT population.

### 2.7 Safety

Analysis of all safety data was performed on the Safety Population and presented descriptively by the treatment received.

## 3 Results

### 3.1 Patient Disposition and Characteristics

A total of 84 subjects were randomized in this study (Figure 1). All of the subjects completed the study and there were no early withdrawals. Two subjects deviated from the protocol by missing a treatment period and 2 subjects had time point window deviations in all TNSS assessments for one period, so they weren't eligible per protocol, but treatment was completed.

**FIGURE 1:**
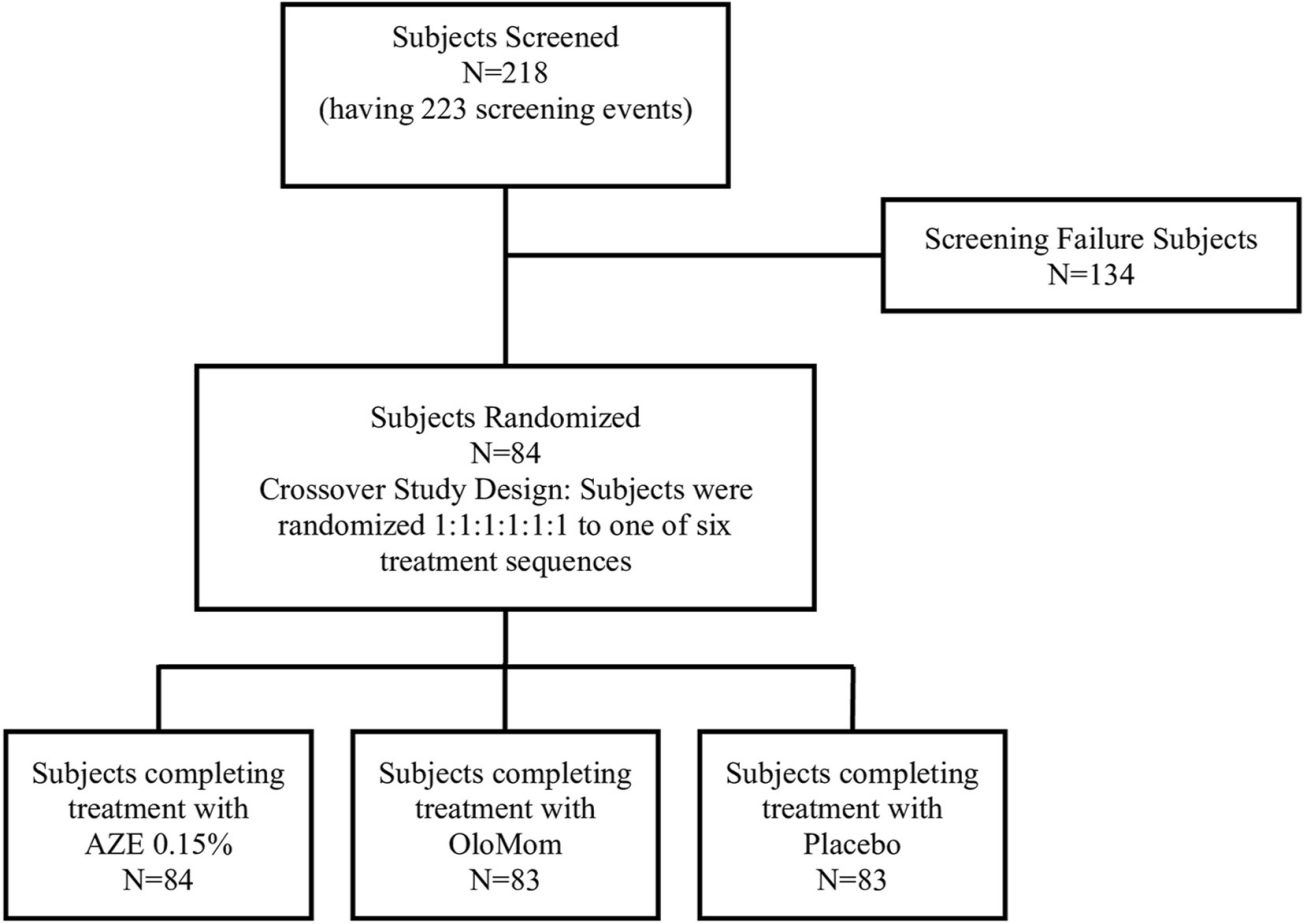
Subject disposition.

The mean age was 41.5 years. The demographic distribution of male and female subjects was similar and included 38 male subjects (45.2%) and 46 female subjects (54.8%). The demographics of the subjects is provided in Supporting Information S1: Table 1. All randomized subjects had a positive skin prick test response to ragweed allergen (a ragweed induced wheal diameter ≥ 3 mm larger than the negative control). No subject had a complete nasal blockage on either or both sides.

### 3.2 Primary Endpoint—Onset of Action

A decrease in allergic rhinitis symptoms (TNSS) was observed in all treatment groups post‐dose. Highest symptom relief was observed in the AZE group throughout the observation period. The onset of action of AZE 0.15% based on the change from baseline in TNSS (LS mean difference ± SE) was 15 min for the ITT population (−1.49 ± 0.259, *p* < 0.0001, Cohen's d: 0.62). The onset of action of OloMom based on the change from baseline in TNSS was also 15 min for the ITT population (−0.68 ± 0.260, *p* = 0.0098, Cohen's d: 0.26), this represents a moderate‐to‐large treatment effect. A statistically significant improvement in TNSS for AZE 0.15% compared to placebo, persisted throughout the entire assessment period (Figure 2). Already after 10 min post‐dose, the difference of 0.39 score points between AZE 0.15% and placebo approached statistical significance (*p* = 0.0864, Cohen's d: 0.21). A maximum difference of −2.26 ± 0.338 for the ITT population was reached at 150 min (*p* < 0.0001, Cohen's d: 0.78).

**FIGURE 2:**
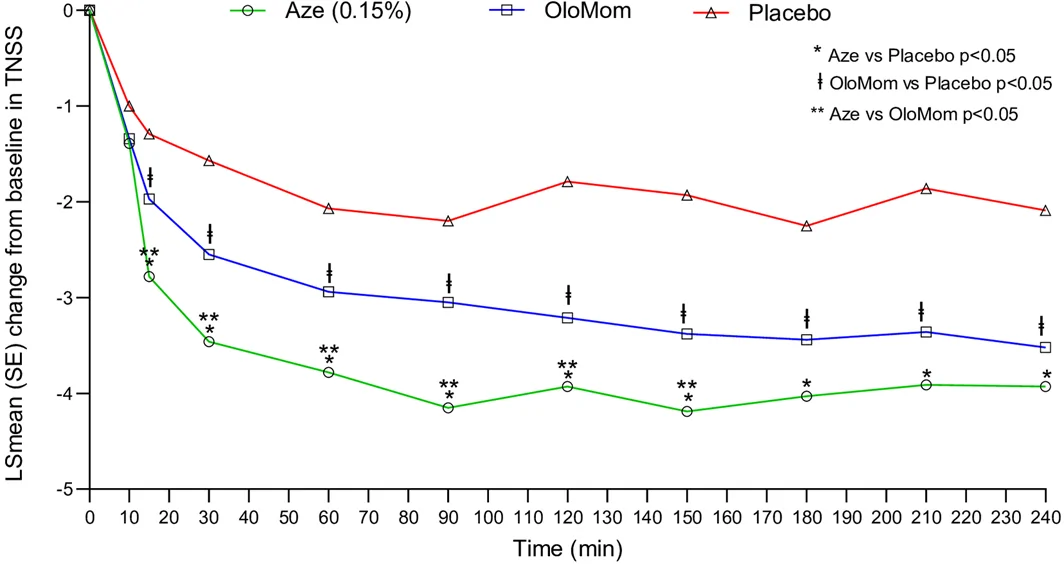
Mean change from baseline in TNSS (0–12) by treatment for 4 h post‐dose (ITT population).

Sensitivity Analysis indicates that AZE 0.15% versus Placebo had an onset for TNSS already at 10 min (−0.62 ± 0.279, *p* = 0.0274) but not for OloMom (−0.38 ± 0.278, *p* = 0.1785). Furthermore, the analysis showed a statistically significant improvement in TNSS for AZE 0.15% compared to placebo, which persisted throughout the entire assessment period. As in the primary analysis, a maximum LS mean difference of −2.32 ± 0.343 (*p* < 0.0001) for the ITT population was reached at 150 min at this sensitivity analysis (Table 1).

**TABLE 1:** LS mean difference between AZE 0.15%, OloMom, and placebo on change from baseline TNSS (ITT population).

	AZE 0.15% versus placebo			OloMom versus placebo			AZE 0.15% versus OloMom		
Time (min)	LS mean (SE)	95% CI	*p*‐value	LS mean (SE)	95% CI	*p*‐value	LS mean (SE)	95% CI	*p*‐value
10	−0.39 (0.226)	−0.84, 0.06	0.0864	−0.33 (0.227)	−0.78, 0.11	0.1430	−0.06 (0.226)	−0.50, 0.39	0.8068
15^a^	−1.49 (0.259)	−2.01, −0.98	< 0.0001	−0.68 (0.260)	−1.20, −0.17	0.0098	−0.81 (0.259)	−1.33, −0.30	0.0020
30	−1.89 (0.280)	−2.44, −1.33	< 0.0001	−0.98 (0.281)	−1.54, −0.43	0.0006	−0.90 (0.279)	−1.46, −0.35	0.0015
60	−1.71 (0.326)	−2.36, −1.07	< 0.0001	−0.87 (0.326)	−1.52, −0.23	0.0083	−0.84 (0.326)	−1.48, −0.20	0.0109
90	−1.95 (0.325)	−2.59, −1.31	< 0.0001	−0.84 (0.328)	−1.49, −0.20	0.0110	−1.11 (0.326)	−1.75, −0.46	0.0009
120	−2.15 (0.313)	−2.76, −1.53	< 0.0001	−1.42 (0.316)	−2.04, −0.80	< 0.0001	−0.72 (0.314)	−1.34, −0.10	0.0222
150	−2.26 (0.338)	−2.93, −1.59	< 0.0001	−1.46 (0.341)	−2.13, −0.78	< 0.0001	−0.80 (0.340)	−1.48, −0.13	0.0192
180	−1.77 (0.345)	−2.45, −1.09	< 0.0001	−1.18 (0.349)	−1.87, −0.49	0.0009	−0.59 (0.347)	−1.27, 0.10	0.0914
210	−2.05 (0.319)	−2.68, −1.42	< 0.0001	−1.50 (0.321)	−2.14, −0.87	< 0.0001	−0.54 (0.321)	−1.18, 0.09	0.0914
240	−1.84 (0.320)	−2.47, −1.21	< 0.0001	−1.44 (0.323)	−2.07, −0.80	< 0.0001	−0.41 (0.321)	−1.04, 0.23	0.2088

*Note:* Negative LS mean differences indicate greater reduction in symptom scores compared with comparator.

^a^ Onset of action.

### 3.3 Secondary Endpoint

#### 3.3.1 Comparison of Onset of Action and Change From Baseline After First Dose, Between AZE 0.15% and OloMom (TNSS, TOSS, and T7SS)

In this study, both AZE 0.15% and OloMom showed a 15‐min onset of action for TNSS versus placebo. However, in sensitivity analysis AZE 0.15% showed onset at 10 minutes (*p* = 0.0274), but not OloMom (*p* = 0.1785) for that onset of action remained unchanged. In a direct comparison, AZE 0.15% demonstrated significantly greater TNSS reduction than OloMom from 15 to 150 minutes (PP and ITT), with the largest difference at 90 minutes (ITT: −1.11 ± 0.326, *p* = 0.0009; PP: −1.18 ± 0.339, *p* = 0.0007).

AZE 0.15% had a shorter time to onset for TOSS (15 min; *p* = 0.0020) than OloMom with the onset at 90 min (*p* = 0.0083, Table 2). Superiority in mean T7SS reduction was seen at 10 min after dosing for AZE 0.15% versus placebo (*p*‐value = 0.05 and 0.02 in ITT and PP analyses, respectively). At the same time point the mean T7SS reduction of OloMom versus Placebo was not statistically significant (*p* = 0.0888 and 0.2616, respectively). Direct comparisons of AZE 0.15% and OloMom show that AZE 0.15% demonstrates a statistically significant larger change from baseline for TOSS at 150 minutes and for T7SS than OloMom from 15 to 150 minutes post‐administration in ITT analysis (*p* value was between 0.002 and 0.02). Maximum LS mean difference between the 2 active treatments was reached at 150 min for TOSS (−0.51 ± 0.205, *p* = 0.0132) and at 90 min for T7SS (−1.45 ± 0.484, *p* = 0.0031). In PP analysis, AZE 0.15% showed larger symptom relief than OloMom for TOSS from 90 – 150 min and 210 – 240 min, and for T7SS from 15 min till the end of the observation period (*p* value was between 0.0007 and 0.045). A maximum LS mean difference between the 2 active treatments was reached at 150 min for TOSS (−0.57 ± 0.206, *p* = 0.0063) and at 90 min for T7SS (−1.62 ± 0.493, *p* = 0.0013).

**TABLE 2:** Onset of action on different total symptom scores (ITT population) of AZE 0.15% and OloMom versus Placebo.

Parameter	Aze versus placebo			OloMom versus placebo		
	Time point (min)	LS mean (SE)	*p*‐value	Time point (min)	LS mean (SE)	*p*‐value
TNSS	15	−1.49 (0.259)	< 0.0001	15	−0.68 (0.260)	0.0098
TNSS (ITT‐ sensitivity analysis)	10	−0.62 (0.279)	0.0274	15	−0.84 (0.292)	0.0042
TOSS	15	−0.53 (0.169)	0.0020	90	−0.56 (0.208)	0.0083
T7SS	15	−2.02 (0.380)	< 0.0001	15	−1.00 (0.382)	0.0098
T7SS (PP‐ sensitivity analysis)	10	−0.83 (0.351)	0.0200	15	−1.09 (0.408)	0.0083

#### 3.3.2 Overall Efficacy (0–4 hours for TNSS, TOSS, and T7SS) After First Dose

AZE 0.15% versus Placebo showed significant differences (both ITT and PP populations) for overall change from baseline in TNSS, TOSS, and T7SS (all *p* < 0.0001). OloMom versus Placebo (both ITT and PP populations) was also significant in TNSS (*p* < 0.0001), TOSS (*p* = 0.0002) and T7SS (*p* < 0.0001) over the complete assessment interval. Although AZE 0.15% has higher effect sizes versus OloMom in both ITT and PP population for overall change from baseline in TNSS, TOSS or T7SS over the complete assessment interval, significant difference for only T7SS was observed in PP population (*p* = 0.0280). AZE 0.15% versus Placebo showed the highest LS mean difference in overall change from baseline in TNSS, TOSS, and T7SS for both ITT [TNSS (−1.02 ± 0.163,*p* < 0.0001); TOSS (−0.51 ± 0.110, *p* < 0.0001); and T7SS (−1.96 ± 0.241, *p* < 0.0001) and PP [TNSS (−1.10 ± 0.155, *p* < 0.0001); TOSS (−0.47 ± 0.112, *p* < 0.0001), and T7SS (−2.04 ± 0.223, *p* < 0.0001)] populations (Table 3).

**TABLE 3:** Overall (0–4 hours) LS mean difference between treatments on change from baseline in TNSS, TOSS, and T7SS (ITT Population) after first‐dose.

Parameter	Comparison	LS mean (SE)	95% CI	*p*‐value
TNSS	AZE 0.15% versus placebo	−1.02 (0.163)	−1.34, −0.70	< 0.0001
	OloMom versus placebo	−0.91 (0.164)	−1.23, −0.59	< 0.0001
	AZE 0.15% versus OloMom	−0.11 (0.163)	−0.43, 0.21	0.4945
TOSS	AZE 0.15% versus placebo	−0.51 (0.110)	−0.73, −0.29	< 0.0001
	OloMom versus placebo	−0.42 (0.111)	−0.64, −0.20	0.0002
	AZE 0.15% versus OloMom	−0.09 (0.111)	−0.31, 0.13	0.4299
T7SS	AZE 0.15% versus placebo	−1.96 (0.241)	−2.44, −1.49	< 0.0001
	OloMom versus placebo	−1.63 (0.242)	−2.10, −1.15	< 0.0001
	AZE 0.15% versus OloMom	−0.34 (0.241)	−0.81, 0.14	0.1655

#### 3.3.3 Overall Efficacy (0–4 Hours for TNSS, TOSS, and T7SS) Post 3‐Day Visit

AZE 0.15% versus Placebo showed significant differences (both ITT and PP populations) for overall change from baseline in TNSS, TOSS, and T7SS (all *p* < 0.0001). Similar results were observed for OloMom versus Placebo in TNSS, TOSS and T7SS over the complete assessment period (all *p* < 0.0001). The differences for ITT between AZE 0.15% and OloMom were not statistically significant for overall change from baseline in TNSS and TOSS over the complete assessment interval (*p* = 0.1158 *p* = 0.2065, respectively). However, T7SS for the ITT population was significant (*p* = 0.0184). For PP population, AZE 0.15% versus OloMom did not show significant difference for overall change from baseline in TNSS (*p* = 0.2929), TOSS (*p* = 0.3531) or T7SS (*p* = 0.0771) (Supporting Information S1: Tables S2 and S3).

#### 3.3.4 Individual Nasal and Ocular Symptoms

The first significant time point for AZE 0.15% versus Placebo for change from baseline in all individual nasal and ocular symptoms was 15 min except for ocular redness (30 minutes).

However, the confirmed onset of action of OloMom was 30 min for runny nose, 60 min for nasal congestion and eye redness, 90 min for itchy nose and itchy eyes, 120 min for sneezing and 150 min for watery eyes. Thus, AZE 0.15% provided faster onset of action than OloMom for all individual nasal and ocular symptoms (Table 4).

**TABLE 4:** Onset of action summary for individual nasal and ocular symptoms after first dose (ITT population).

Parameter	AZE 0.15% versus placebo			OloMom versus placebo		
	Time point (min)	LS mean (SE)	*p*‐value	Time point (min)	LS mean (SE)	*p*‐value
Itchy nose	15	−0.42 (0.087)	< 0.0001	90	−0.21 (0.098)	0.0328
Nasal congestion	15	−0.35 (0.085)	< 0.0001	60	−0.23 (0.095)	0.0156
Runny nose	15	−0.34 (0.083)	< 0.0001	30	−0.21 (0.090)	0.0222
Sneezing	15	−0.39 (0.094)	< 0.0001	120	−0.33 (0.110)	0.0037
Itchy eyes	15	−0.27 (0.078)	0.0007	90	−0.23 (0.093)	0.0169
Watery eyes	15	−0.16 (0.075)	0.0316	150	−0.20 (0.092)	0.0305
Eye redness	30	−0.24 (0.069)	0.0008	60	−0.22 (0.076)	0.0042

Also, direct comparisons of AZE 0.15% and OloMom showed that AZE 0.15% demonstrated a larger change from baseline from 15 to 210 min post‐administration for sneezing and occasionally for other symptoms. Overall efficacy analyses confirmed effectiveness of both active treatments over Placebo in relief of all individual nasal and ocular symptoms.

### 3.4 Safety

Five subjects experienced 6 TEAEs during the study. Two subjects reported one TEAE each after receiving Azelastine. Both TEAEs (somnolence and dysgeusia) were reported as related for Azelastine. One subject reported 2 TEAEs (alanine aminotransferase increased and blood alkaline phosphatase increased) after the use of OloMom and 2 subjects reported 2 TEAEs (both are headache) after using Placebo treatment. The majority (5/6) of the TEAEs were mild in severity with only 1 TEAE (somnolence) graded as moderate. No serious or severe TEAEs were reported in this study and no subjects discontinued from the study or study treatment due to a TEAE. No deaths were reported during the study.

## 4 Discussion

Patients with AR often prefer rapid‐onset treatments; intranasal corticosteroids or antihistamines are commonly used on‐demand for quick symptom relief [15]. This treatment scheme offers flexibility and reduces overall medication use, which can be beneficial for patient adherence and minimizing side effects. Although INS have a well‐established safety profile, steroid phobia is a significant barrier in the treatment of AR [16]. In addition, INS have a slower onset of action compared to antihistamines [6]. On‐demand treatment of AR using azelastine has shown to be effective for rapid symptom relief. [17]. Although previous controlled studies using EEU showed that AZE 0.10% provided symptom relief within 15 minutes, in a recent trial using the same methodology AZE 0.15% became effective within 30 min [12]. However, the latter study was conducted during the COVID‐19 pandemic and was affected by numerous operational challenges, resulting in a high number of study discontinuations. Standard Phase 3 efficacy studies for AR evaluated effect under clinical conditions [4] but the primary endpoint was to determine treatment effect over 2‐week treatment, and they were not particularly designed to detect early onset of action. OloMom demonstrated a significant TNSS improvement versus placebo (*p* = 0.02) at 10 minutes after administration when evaluated in ragweed pollen in an EEC model. However, the superiority over placebo was not maintained at all time points (i.e., *p* = 0.06 at 2.5 hours) in the 4‐h assessment period [14].

In this study, AZE 0.15% showed significant TNSS reduction versus placebo from 15 minutes onward, with sensitivity analysis excluding out‐of‐window assessments indicating onset as early as 10 minutes that was faster than OloMom. Since the actual timing of symptom score assessments is critical for evaluating the onset of action, this sensitivity analysis is considered particularly appropriate. AZE 0.15% demonstrated greater early TNSS improvement compared with OloMom, with differences diminishing at later time points. At 15‐min timepoint, strong evidence of efficacy with moderate treatment effect was seen with AZE 0.15% but not with OloMom confirmed by larger treatment differences to placebo (−1.49 ± 0.259, *p* < 0.0001 vs. −0.68 ± 0.260, *p* = 0.0098) and a higher effect size (Cohen's *d* = 0.62 vs. 0.26). Sensitivity analysis excluding out‐of‐window data confirmed AZE's faster and more reliable symptom relief.

AZE 0.15% showed more advantages over OloMom in fast relief of ocular symptoms. Furthermore, relief of individual nasal and ocular symptoms began promptly after the administration of AZE 0.15% (15 minutes except for eye redness it was 30 min), demonstrating a consistent and rapid effect across all symptoms. Relief individual nasal and ocular symptoms began slower with OloMom with the onset of action of ranging from 30 minutes for runny nose to 150 minutes for watery eye.

Azelastine and olopatadine are both second‐generation antihistamines used to treat allergic conditions, but they have distinct mechanisms of action. Azelastine works by blocking histamine H1 receptors and stabilizing mast cells, thereby inhibiting the release of histamine and other inflammatory mediators such as leukotrienes and cytokines [18]. Olopatadine also blocks histamine H1 receptors and acts as a mast cell stabilizer, preventing the release of histamine and pro‐inflammatory cytokines [19]. While both medications are effective in managing allergic symptoms, azelastine may have a broader range of action due to its additional inhibition of leukotrienes and cytokines. Azelastine's ability to block histamine H1 receptors, stabilize mast cells, and inhibit the release of leukotrienes and cytokines makes it highly effective for rapid and comprehensive symptom relief [18]. Clinical studies have shown that intranasal azelastine provides rapid relief from nasal symptoms such as rhinorrhea, sneezing, nasal congestion, and nasal pruritus [19]. The present study confirmed the rapid onset of action with AZE 0.15% not only in relief of nasal but also in relief of ocular symptoms and that its onset was faster than with OloMom.

Current practice guidelines recommend intranasal antihistamines (INAHs) as the first line treatment for either mild or moderate‐to‐severe AR [20]. For persistent AR, intranasal corticosteroids (INCS) are the first line therapy, subsequent to oral antihistamines and INAHs, for mild symptoms, and use of both INAHs and INCS for moderate/severe symptoms [21]. The rapid onset of INAHs offers an advantage compared to an onset of 1–3 hours for oral antihistamines and many hours or even days for INCS [22, 23]. Nasal congestion is a specifically common and bothersome symptom of AR. Even though decongestants are another effective and commonly used treatment, they only relieve nasal congestion and are limited in use for 3–5 days. The current study shows that AZE 0.15% was quick and effective on all nasal and ocular symptoms of AR. There is consistency in efficacy among the ITT and PP population. In addition, the present study showed that following 3‐day treatment, AZE 0.15% had similar efficacy as OloMom and could match the treatment behavior shown by patients of using medications only short‐term when being symptomatic. Both AZE 0.15% and OloMom were safe and well‐tolerated.

## 5 Conclusion

AZE 0.15% demonstrated an onset of action on TNSS as early as 15 min like OloMom but with strong evidence of efficacy and larger effect size. Additionally, sensitivity analyses excluding out‐of‐window assessments supported a faster onset of action at 10 min on TNSS for AZE 0.15%. AZE 0.15% had an earlier time to onset of action for TOSS (15 minutes) as compared to OloMom (TOSS 90 minutes). For all individual nasal and ocular symptoms AZE 0.15% provided faster relief confirmed by earlier time to onset of action. After 3.5 days of treatment the overall efficacy in alleviating nasal (TNSS) and ocular symptoms (TOSS) was comparable between AZE 0.15% and OloMom. AZE 0.15% had similar efficacy compared to OloMom over the entire treatment period and could be alternative steroid free option for rapid symptom control in children and patients with pollen allergies and corticophobia.

## Author Contributions

**Jean Bousquet:** conceptualization, writing – review and editing, validation, writing – original draft. **Ludger Klimek:** conceptualization, writing – original draft, writing – review and editing. **Hans‐Christian Kuhl:** methodology, software. **Duc Tung Nguyen:** writing – review and editing, methodology, data curation. **Rajesh Kumar:** methodology, writing – original draft, validation. **William Berger E:** conceptualization, writing – original draft, writing – review and editing.

## Funding

This study was funded by Viatris.

## Ethics Statement

The study was approved by the Canadian competent authority (Health Canada) in agreement with the following laws and guidelines: (a) Declaration of Helsinki (G2). (b) ICH guideline on Good Clinical Practice (ICH E6 (R2)) Addendum (G3). (c) Regulation (EU) 2016/679 of 27 April 2016 (General Data Protection Regulation (G4)). (d) Applicable regulatory and national legislations. Cliantha Research applied for authorization by competent authorities, as applicable, and for favorable opinion by the Advarra IRB, registered with OHRP and FDA under IRB#00000971. MEDA provided a power of attorney for delegation of responsibilities to authorized persons/institutions. The report is in accordance with the current ICH E3, Guideline for Industry, Structure and Content of Clinical Study Reports.

## Conflicts of Interest

Hans‐Christian Kuhl, Duc Tung Nguyen and Rajesh Kumar are employees of Viatris. All other authors declare no conflicts of interest associated with the publication of this study.

## Supporting information


Supporting Information S1

## Data Availability

The data supporting the findings of this study are included within the article and its supplementary information files. No additional data is available.
