# Supplementary material for: Onset of Action of Azelastine 0.15% Versus Olopatadine/Mometasone for Allergen‐Induced Rhinitis: An EEU Crossover Study
Source: Clin Transl Allergy. 2026 Nov 8;16(11):e70213. doi: 10.1002/clt2.70213 (PMC13745312; doi:10.1002/clt2.70213)
Supplement: Supplementary file 1 [file CLT2-16-e70213-s001.docx]

**Supplementary Table 1: Subject demographics**

| **Parameter** | **Category** | **ITT**  **(N=84)** | **PP**  **(N=80*)** |
| --- | --- | --- | --- |
| Sex, n(%) | Male | 38 (45.2) | 37 (46.3) |
|  | Female | 46 (54.8) | 43 (53.8) |
| Age  (Completed Years) | Mean (SD) | 41.5 (8.64) | 41.5 (8.73) |
|  | Median | 43.0 | 43.0 |
| Race | American Indian or Alaska Native | 0 (0) | 0 (0) |
|  | Asian | 16 (19.0) | 15 (18.8) |
|  | Black or African American | 21 (25.0) | 19 (23.8) |
|  | Native Hawaiian or Other Pacific Islander | 1 (1.2) | 1 (1.3) |
|  | White | 44 (52.4) | 43 (53.8) |
|  | Other | 2 (2.4) | 2 (2.5) |
| Ethnicity (n (%)) | Hispanic or Latino | 15 (17.9) | 14 (17.5) |
|  | Not Hispanic or Latino | 69 (82.1) | 66 (82.5) |
|  | Not reported | 0 (0) | 0 (0) |
|  | Unknown | 0 (0) | 0 (0) |
|  | Other | 0 (0) | 0 (0) |
| Height (cm) | Mean (SD) | 169.9 (10.47) | 170.2 (10.25) |
|  | Median | 171.0 | 171.0 |
| Weight (kg) | Mean (SD) | 77.99 (17.460) | 78.28 (17.449) |
|  | Median | 75.35 | 75.35 |
| BMI (kg/m^2^) | Mean (SD) | 26.91 (4.905) | 26.93 (4.923) |
|  | Median | 26.65 | 26.65 |

Supplementary Table 2: Overall (0-4 hours) LS Mean Difference between Treatments on Change from Baseline in TNSS, TOSS, and T7SS post 3-day visit (PP Population)

| **Parameter** | **Comparison** | **LS Mean (SE)** | **95% CI** | **p-value** |
| --- | --- | --- | --- | --- |
| **TNSS** | AZE 0.15% vs Placebo | -1.76 (0.233) | -2.22, -1.30 | <0.0001 |
|  | OloMom vs Placebo | -2.01 (0.235) | -2.47, -1.54 | <0.0001 |
|  | AZE 0.15% vs OloMom | 0.25 (0.234) | -0.22, 0.71 | 0.2929 |
| **TOSS** | AZE 0.15% vs Placebo | -1.10 (0.155) | -1.40, -0.79 | <0.0001 |
|  | OloMom vs Placebo | -1.24 (0.155) | -1.55, -0.94 | <0.0001 |
|  | AZE 0.15% vs OloMom | 0.14 (0.155) | -0.16, 0.45 | 0.3531 |
| **T7SS** | AZE 0.15% vs Placebo | -3.01 (0.359) | -3.72, -2.30 | <0.0001 |
|  | OloMom vs Placebo | -3.65 (0.360) | -4.37, -2.94 | <0.0001 |
|  | AZE 0.15% vs OloMom | 0.64 (0.359) | -0.07, 1.35 | 0.0771 |

Supplementary Table 3: Overall (0-4 hours) LS Mean Difference between Treatments on Change from Baseline in TNSS, TOSS, and T7SS post 3-day visit (ITT Population)

| **Parameter** | **Comparison** | **LS Mean (SE)** | **95% CI** | **p-value** |
| --- | --- | --- | --- | --- |
| **TNSS** | AZE 0.15% vs Placebo | -1.73 (0.253) | -2.23, -1.23 | <0.0001 |
|  | OloMom vs Placebo | -2.13 (0.254) | -2.63, -1.62 | <0.0001 |
|  | AZE 0.15% vs OloMom | 0.40 (0.253) | -0.10, 0.90 | 0.1158 |
| **TOSS** | AZE 0.15% vs Placebo | -1.03 (0.154) | -1.33, -0.72 | <0.0001 |
|  | OloMom vs Placebo | -1.22 (0.155) | -1.53, -0.92 | <0.0001 |
|  | AZE 0.15% vs OloMom | 0.20 (0.154) | -0.11, 0.50 | 0.2065 |
| **T7SS** | AZE 0.15% vs Placebo | -2.68 (0.366) | -3.40, -1.95 | <0.0001 |
|  | OloMom vs Placebo | -3.55 (0.368) | -4.28, -2.82 | <0.0001 |
|  | AZE 0.15% vs OloMom | 0.87 (0.367) | 0.15, 1.60 | 0.0184 |
